# Epitranscriptomic 5-Methylcytosine Profile in PM_2.5_-induced Mouse Pulmonary Fibrosis

**DOI:** 10.1016/j.gpb.2019.11.005

**Published:** 2020-03-03

**Authors:** Xiao Han, Hanchen Liu, Zezhong Zhang, Wenlan Yang, Chunyan Wu, Xueying Liu, Fang Zhang, Baofa Sun, Yongliang Zhao, Guibin Jiang, Yun-Gui Yang, Wenjun Ding

**Affiliations:** 1 College of Life Sciences, University of Chinese Academy of Sciences, Beijing 100049, China; 2 CAS Key Laboratory of Genomic and Precision Medicine, Collaborative Innovation Center of Genetics and Development, College of Future Technology, Beijing Institute of Genomics, Chinese Academy of Sciences, Beijing 100101, China; 3 Sino-Danish College, University of Chinese Academy of Sciences, Beijing 101408, China; 4 Research Center for Eco-Environmental Sciences, Chinese Academy of Sciences, Beijing 100085, China; 5 Institute of Stem Cell and Regeneration, Chinese Academy of Sciences, Beijing 100101, China

**Keywords:** PM_2.5_ exposure, mRNA m^5^C, Pulmonary fibrosis, Inflammation, Immune response

## Abstract

Exposure of airborne particulate matter (PM) with an aerodynamic diameter less than 2.5 μm (PM_2.5_) is epidemiologically associated with lung dysfunction and respiratory symptoms, including **pulmonary fibrosis**. However, whether epigenetic mechanisms are involved in PM_2.5_-induced pulmonary fibrosis is currently poorly understood. Herein, using a PM_2.5_-induced pulmonary fibrosis mouse model, we found that **PM_2.5_ exposure** leads to aberrant mRNA 5-methylcytosine (m^5^C) gain and loss in fibrotic lung tissues. Moreover, we showed the m^5^C-mediated regulatory map of gene functions in pulmonary fibrosis after PM_2.5_ exposure. Several genes act as m^5^C gain-upregulated factors, probably critical for the development of PM_2.5_-induced fibrosis in mouse lungs. These genes, including *Lcn2*, *Mmp9*, *Chi3l1*, *Adipoq*, *Atp5j2*, *Atp5l, Atpif1*, *Ndufb6*, *Fgr*, *Slc11a1*, and *Tyrobp*, are highly related to oxidative stress response, inflammatory responses, and immune system processes. Our study illustrates the first epitranscriptomic RNA m^5^C profile in PM_2.5_-induced pulmonary fibrosis and will be valuable in identifying biomarkers for PM_2.5_ exposure-related lung pathogenesis with translational potential.

## Introduction

Exposure to airborne particulate matter with aerodynamic diameter less than 2.5 μm (PM_2.5_) has been epidemiologically associated with respiratory diseases [1], [2]. Harmful PM_2.5_ pollutants are released into the pulmonary surfactant and then attached to pulmonary epithelial cells [2]. Thus, it can increase the risk of multiple airway illnesses, including chronic obstructive pulmonary disease (COPD) [3], bronchitis [4], asthma [5], and idiopathic pulmonary fibrosis (IPF) [6]. Pulmonary fibrosis is characterized by excessive deposition of collagen in the lungs, leading to chronically impaired gas exchange and death [6]. It has been verified that PM_2.5_ and PM_10_ exposure accelerates functional decline in IPF patients determined by linear multivariable mixed-effects model [7].

Inflammatory responses [8], [9], immune cell activation [9], [10], [11], and oxidative stress [12], [13] are highly associated with pulmonary fibrosis pathogenesis. Oropharyngeal aspiration of PM_2.5_ induces significant collagen deposition and increases in the levels of the inflammatory markers interleukin 1-β (IL-1β) and transforming growth factor (TGF-β1), in mouse lungs after 21 days of treatment [8]. Anti-inflammatory cytokine IL-13 mediates fibrogenesis by regulating TGF-β1 expression and recruiting leukocytes into the lesion site [9]. Tumor necrosis factor-α (TNF-α) can also activate macrophages to secrete TGF-β1, leading to extracellular matrix (ECM) deposition [11]. In addition, high levels of oxidant stress have been detected in IPF patients compared with controls [14], and oxidant–antioxidant imbalances in the lower respiratory tract are strongly associated with IPF pathogenesis [12]. A recent study has unraveled that exposure to PM_2.5_ promotes fibrotic response by increasing the expression of Col1a1, Col3a1, Cox-4, and TGF-β1, activating *smad3* expression, as well as generating reactive oxygen species (ROS) [15]. These findings further support the critical role of oxidative stress in fibrogenesis.

Epigenetic mechanisms, especially aberrant transcriptome induced by altered DNA methylation, have been shown to be associated with PM_2.5_ exposure and possible pathogenesis [16], [17]. RNA methylation, another epigenetic mechanism that regulates gene expression, has been shown to participate in various disease processes [18]. For example, as the most abundant internal RNA modification, *N*^6^-methyladenosine (m^6^A) is involved in acute myelocytic leukemia (AML), metabolic disorders, and nervous system disorders [18]. 5-Methylcytosine (m^5^C) is another prevalent RNA modification in mammals [19], [20] and plants [21], [22]. m^5^C modification is catalyzed by NOP2/Sun RNA methyltransferase family member 2 (NSUN2) in humans, mice, and human immunodeficiency viruses (HIV) [19], [20], [23], [24], [25], [26]. NUSN2 has been shown to mediate RNA transport by m^5^C reader of the nuclear protein Aly/REF export factor (ALYREF) [20], [27]. m^5^C participates in the regulation of RNA processing [24], [28], RNA stability [29], [30], [31], [32], and translation [33], [34]. Moreover, it plays critical roles in several important biological pathways in mice and plants [19], [22]. Recent advances in high-throughput sequencing technologies have facilitated m^5^C site identification at single-base resolutions [19], [20], [22], [23]. These findings suggest that m^5^C modification serves as a key posttranscriptional regulatory factor. However, whether the m^5^C methylome is altered upon PM_2.5_ exposure and further participates in the pathogenesis of pulmonary fibrogenesis is not well understood.

In our previous study, we have uncovered that PM_2.5_ directly affects macrophage polarization and induces the expression of the pro-inflammatory cytokines granulocyte–macrophage colony stimulating factor (GM-CSF), TNF-β, IL-1β, and IL-6 in mice [10]. In this study, the m^5^C methylome was profiled in the pathogenesis of lung fibrosis. We identified a possible novel role of altered m^5^C modification in mediating PM_2.5_-induced lung fibrosis through posttranscriptional regulation.

## Results and discussion

### PM_2.5_ exposure leads to pulmonary inflammation and fibrosis

**Figure 1:**
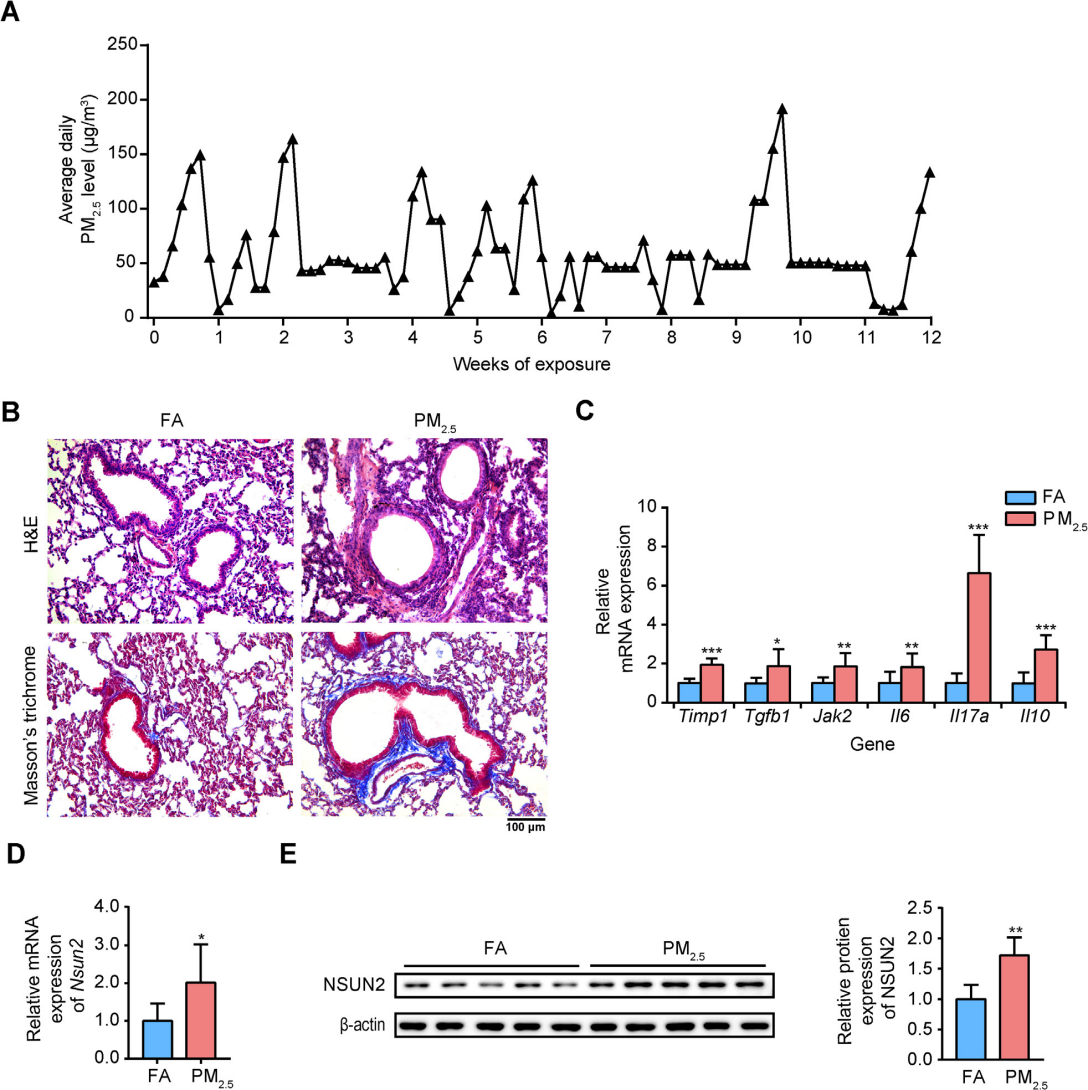
**PM_2.5_ exposure induced pulmonary inflammation and fibrosis** **A.** The average daily PM_2.5_ concentrations in the exposure chamber during the study period. **B**. Representative images of H&E and Masson’s trichrome staining in lungs of FA- and PM_2.5_-exposed mice. Scale bar = 100 μm. **C.** RT-qPCR analysis showing the relative expression levels (compared to FA) of inflammatory and fibrotic genes in mouse lungs. **D.** The mRNA expression of *Nsun2* in mouse lungs examined by RT-qPCR. **E.** The protein expression of NSUN2 in mouse lungs examined by Western blotting. The *P* values were determined using two-tailed Student’s *t*-tests. *, *P* < 0.05; **, *P* < 0.01; ***, *P* < 0.01. FA, filtered air; PM_2.5_, particulate matter with an aerodynamic diameter less than 2.5 μm; H&E, hematoxylin and eosin; Timp1, tissue inhibitor of metalloproteinase 1; Tgfb1, transforming growth factor, beta 1; Jak2, Janus kinase 2; Il, interleukin; NSUN, NOL1/NOP2/Sun domain family protein.

To mimic human exposure as accurately as possible, mice were maintained in ambient airborne PM_2.5_ chamber (Table S1). As shown in Figure 1A, the weekly average PM_2.5_ concentrations during the exposure period varied from 35.66 μg/m^3^ to 101.60 μg/m^3^. The mean airborne PM_2.5_ concentration to which the mice were exposed during the study period was ∼59.77 μg/m^3^. Histopathological examination of lung sections using hematoxylin and eosin (H&E) and Masson staining showed that compared to filter air (FA)-exposed mice, PM_2.5_-exposed mice exhibited severe lung injury and fibrosis (Figure 1B). Exposure caused intense inflammatory cell infiltration and thickened alveolar walls in the lungs (Figure 1B, top); it also caused excessive collagen deposition around the bronchi (Figure 1B, bottom). Moreover, the expression levels of the inflammatory markers *Timp1*, *Tgfb1*, *Jak2*, *Il6*, *Il17a*, and *Il10* were higher in the PM_2.5_-exposed group than control group (*P* < 0.05, two-sided Wilcoxon and Mann–Whitney tests, Figure 1C). These results are consistent with previous reports demonstrating that PM_2.5_-induced disorder of inflammatory cytokine networks may lead to the death of lung epithelial cells and fibroblasts [35], [36], [37]. Importantly, we found that PM_2.5_ exposure increased NSUN2 mRNA and protein levels in the lung (Figure 1D and E). NSUN2 has been shown to methylate a variety of mRNAs and to promote or inhibit cell growth and proliferation [38]. It has been reported that the protein expression of NSUN2 is increased in human breast cancer [39]. As NSUN2 is a well-validated m^5^C methyltransferase [19], [20], [23], [24], [25], it is possible that altered expression of *Nsun2* could disturb basic biological functions by affecting the number or methylation level of m^5^C sites. Thus, we hypothesize that PM_2.5_ may contribute to altered mRNA m^5^C methylomes during pulmonary fibrosis development.

### PM_2.5_ exposure triggers m^5^C modification in pulmonary fibrosis

**Figure 2:**
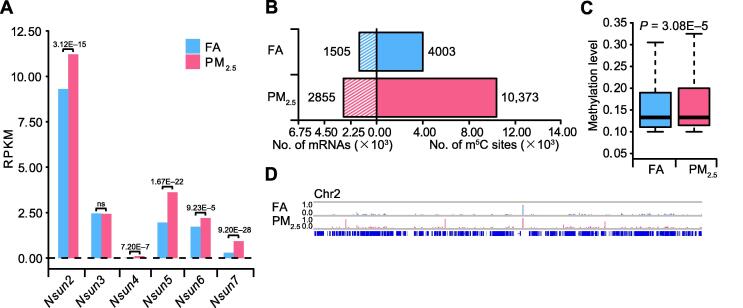
**Distribution profile of m^5^C in the lung tissues of FA- and PM2.5-exposed mice** **A.** Bar chart illustrating the expression levels of NOP2/Sun RNA methyltransferase family protein members in lungs of FA- and PM_2.5_-exposed mice, as examined by RNA-Seq. FDR values calculated using Benjamini–Hochberg method were labeled on top of the genes. **B.** Bar chart showing the numbers of m^5^C sites and m^5^C-modified mRNAs lung samples of FA- and PM_2.5_-exposed mice. **C.** Boxplots showing the overall distributions of mRNA m^5^C levels in lung samples of FA- and PM_2.5_-exposed mice using meRanTK. The *P* values were calculated using two-sided Wilcoxon and Mann–Whitney tests. **D.** IGV tracks displaying the distributions of m^5^C methylation level (in the range of 0.0–1.0) on chromosome 2 in lung samples of FA- and PM_2.5_-exposed mice. FDR, false positive rate; IGV, Integrative Genomics Viewer.

To define the potential mechanism of PM_2.5_-induced pulmonary fibrosis, we performed RNA bisulfite sequencing (RNA-BisSeq) and RNA sequencing (RNA-Seq) for the lung samples from FA- or PM_2.5_-exposed mice (Table S2). We found that the mRNA levels of most *Nsun* family members were upregulated in lung samples from PM_2.5_-exposed group with fold change (FC) > 1.2 and false positive rate (FDR) < 9.23E−5. Of all the *Nsuns* members, *Nsun2* showed the highest expression level (Figure 2A). These results are consistent with the results of Western blot and RT-qPCR analyses (Figure 1D and E). Similarly, we observed that expression of *Sftpc*, *Lcn2*, *Dbp*, *Mmp9*, *Ace2*, and *Hist1h2ad* was upregulated in PM_2.5_-induced pulmonary fibrosis, whereas expression of *Pink1*, *Jak1*, *Tgfb*, and other nine pulmonary fibrosis-related genes was downregulated (Figure S1A) [40], [41], [42], [43]. Interestingly, 5/6 of the upregulated genes (*Lcn2*, *Dbp*, *Mmp9*, *Ace2*, and *Hist1h2ad*) had more m^5^C sites when comparing ling samples from PM_2.5_-exposed to those from FA-exposed mice samples. The results support a reasonable speculation that m^5^C probably participates in the regulation of pulmonary fibrosis development.

NSUN2 upregulation is probably the main reason for m^5^C alterations. It has been demonstrated that PM_2.5_ can elicit oxidative stress by inducing ROS generation and disrupting intracellular redox balance [17]. Moreover, oxidative stress (H_2_O_2_ exposure) has been shown to increase NSUN2 protein expression [44]. We found that transcription factors (TFs) encoded by 12 genes, including *Egr1*, *Eg*r2, *Hlf*, *Hoxa3*, *Hsf1*, *Hsf2*, *Irf7*, *Max*, *Nfe2*, *Pax6*, *Pparg*, and *Stat4* (*P* < 0.001, hypergeometric test), can potentially bind to the promoter sequence of *Nsun2* [45]. Expression of these genes was upregulated in PM_2.5_-exposed group (FC > 1.2, FDR < 0.05). Thus, we speculate that PM_2.5_ induces oxidative stress and increases expression of TFs, which are involved in the upregulation of *Nsun2* and consequently the increase in m^5^C level. However, how PM_2.5_ induces pulmonary fibrogenesis through modulating NSUN2 and TFs warrants further investigation.

**Figure 3:**
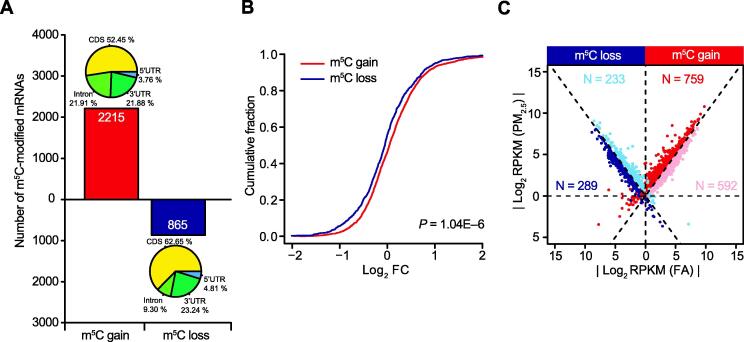
**mRNA m^5^C gain or loss is associated with aberrant transcriptome** **A.** Number of mRNAs with m^5^C gain (red) and loss (dark blue) in lung samples from PM_2.5_-exposed mice, compared to those from FA-exposed mice. The pie chart shows the distributions of m^5^C sites in different genic regions. **B.** Cumulative distribution displaying the expression level change of genes with m^5^C gain (red) and loss (dark blue) from RNA-Seq data in lung samples from PM_2.5_-exposed mice, compared to those from FA-exposed mice. The *P* values were calculated using two-sided Wilcoxon and Mann–Whitney tests and are shown in the bottom-right corner. **C.** Scatter plot showing the distributions of genes with significant RNA abundance changes (FC > 1.5, FDR < 0.05) with regard to both m^5^C gain/loss and gene expression levels in lung samples from PM_2.5_-exposed mice, compared to those from FA-exposed mice. The dots in red, pink, blue, and dark blue represent upregulated genes with m^5^C gain, downregulated genes with m^5^C gain, upregulated genes with m^5^C loss and downregulated genes with m^5^C loss, respectively. FC, fold change.

We next compared the m^5^C features between PM_2.5_-exposed and control FA groups. We found consistent m^5^C distribution in different regions and conserved m^5^C motif (Figure S1B and C). Intriguingly, the numbers of m^5^C sites and modified mRNAs increased markedly in PM_2.5_-treated group; 6370 additional m^5^C sites in 1350 mRNAs were detected in the PM_2.5_-treated group (Figure 2B). The global methylation level was also increased (*P* = 3.08E−5, two-sided Wilcoxon and Mann–Whitney tests, Figure 2C). In addition, we examined the number of modified mRNAs among different chromosomes. Almost all the chromosomes underwent m^5^C gain, except mitochondrial and Y chromosomes, most likely due to their shorter genome lengths (Figure S1D). The Integrative Genomics viewer (IGV) tracks also displayed a wide range of m^5^C gain across the whole chromosome 2 (Figure 2D). We then identified 2215 mRNAs with m^5^C gain and 865 mRNAs with m^5^C loss in lungs of PM_2.5_-exposed mice compared to those of FA-exposed mice (Figure 3A). The expression of genes with m^5^C gain was upregulated compared to that of genes with m^5^C loss overall (*P* = 1.04E−6, two-sided Wilcoxon and Mann–Whitney tests, Figure 3B). To exclude the potential impact caused by gene expression, we compared the coverage of m^5^C-modified genes based on the RNA-BisSeq data for the m^5^C gain and loss groups between lung samples of FA and PM_2.5_-exposed mice RNA-BisSeq (Figure S2A). High Pearson correlation coefficients between FA- and PM_2.5_-exposed mice for gene coverage were obtained in both the m^5^C gain (*R* = 0.89, *P* < 2.20E−16) and loss groups (*R* = 0.99, *P* < 2.20E−16). This result indicates that the m^5^C global changes are not affected by gene expression.

We further identified 12,595 differentially expressed genes (DEGs) in PM_2.5_-exposed group (FC > 1.2, FDR < 0.05), including 8964 upregulated DEGs and 3631 downregulated DEGs (Figure S2B). Functional enrichment analysis showed that these DEGs were highly enriched in cellular metabolic processes, RNA processing, immune responses, anatomical structure morphogenesis, system development, and cell differentiation (Figure S2C and D). To delineate the impact of mRNA m^5^C on the transcriptome profile in mouse pulmonary fibrosis, we compared the alterations in RNA abundance of gene sets with m^5^C gain or loss. We divided the gene sets into four groups (see details in Materials and methods): upregulated genes with m^5^C gain (*n* = 759), upregulated genes with m^5^C loss (*n* = 233), downregulated genes with m^5^C gain (*n* = 592), and downregulated genes with m^5^C loss (*n* = 289) (Figure 3C). Importantly, over half of the genes with m^5^C gain exhibited upregulated expression levels (759 out of 1351, 56.18%). We found hundreds of mRNAs whose expression was abundant and probably affected by m^5^C gain or loss in lungs of PM_2.5_-exposed mice. Altogether, m^5^C modification may regulate expression of the modified genes that are associated with the pathogenesis of PM_2.5_-induced pulmonary fibrosis.

### m^5^C modification regulates PM_2.5_-induced immune and fibrotic responses

**Figure 4:**
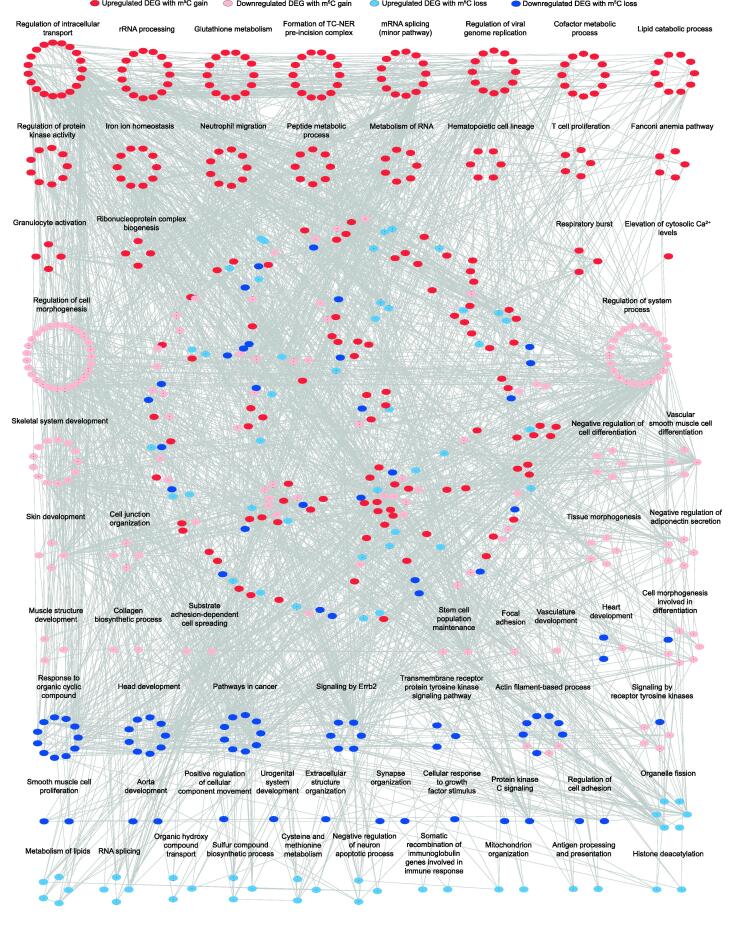
**GO enrichment map of m^5^C-modifed DEGs** Groups of upregulated genes with m^5^C gain (red), downregulated genes with m^5^C gain (pink), upregulated genes with m^5^C loss (blue), and downregulated genes with m^5^C loss (dark blue) are marked in the network (*P* < 0.01, hypergeometric test; for details, see Tables S3 and S4). The dots in the middle of the network with no functional labels represent genes that participate in multiple biological processes. GO, Gene Ontology; PPI, protein–protein interaction; DEG, differentially expressed gene.

To further determine the biological significance of the m^5^C-modified DEGs in the lungs of mice exposed to PM_2.5_, gene ontology (GO) and pathway analyses were carried out. A protein–protein interaction (PPI) network analysis of the proteins encoding these DEGs indicates that both up- and downregulated DEGs are involved in multiple processes (Figures 4 and S3; Tables S3 and S4). As shown in Figure 4, for example, upregulated genes with m^5^C gain were enriched in neutrophil migration, granulocyte activation, as well as mRNA splicing and metabolism. In contrast, downregulated genes with m^5^C loss were involved in cancer, cell adhesion regulation, and other processes. These results suggest that PM_2.5_ exposure may regulate mRNA processing in lung lesions, leading to the recruitment and activation of immune cells. In particular, mRNA m^5^C gain negatively affected normal metabolic activity by upregulating expression levels of some genes in the lung. Therefore, we speculate that mRNA m^5^C modification may function as a pivotal factor regulating the pathogenesis of pulmonary fibrosis.

**Figure 5:**
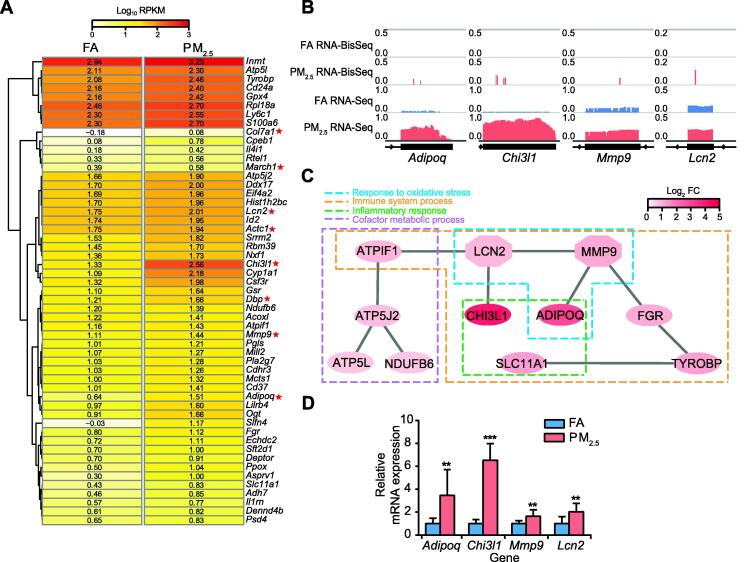
**Upregulated DEGs with m^5^C gain were associated with immune and inflammatory responses in pulmonary fibrosis** **A.** Heatmap illustrating the expression levels of the top 53 upregulated DEGs with m^5^C gain (ranked by FDR) in mouse lungs (FC > 1.5, FDR < 0.05). The red asterisks mark the important DEGs with validated functions in oxidative stress responses, inflammatory responses, and immune system processes. **B.** IGV tracks displaying m^5^C and RNA-Seq read distributions in the FA (blue) and PM_2.5_-exposed (watermelon red) samples (from left to right: *Adipoq*, *Chi3l1*, *Mmp9*, and *Lcn2*). **C.** PPI network map of 11 pulmonary fibrosis-related upregulated genes with m^5^C gain. The color bar represents the mRNA log_2_ fold change between the two groups. **D.** RT-qPCR results showing the increased expression of four representative genes in lung samples from FA-exposed (bars in blue) and PM_2.5_-exposed (bars in watermelon red) mice. *P* values were determined using two-tailed Student’s *t*-tests. *, *P* < 0.05; **, *P* < 0.01; ***, *P* < 0.001. ADOPOQ, adiponectin, C1Q and collagen domain-containing; CHI3L1, chitinase-like 1; MMP9, matrix metallopeptidase 9; LCN2, lipocalin 2; ATPIF1, ATPase inhibitory factor 1; ATP5J2, ATP synthase, H^+^-transporting mitochondrial F0 complex subunit F2; ATP5L, ATP synthase, H^+^-transporting mitochondrial F0 complex subunit G; NDUFB6, NADH: ubiquinone oxidoreductase subunit B6; SLC11A1, solute carrier family 11; TYROBP, TYRO protein tyrosine kinase binding protein.

GO enrichment map of these gene sets showed that upregulated genes with m^5^C gain were mainly associated with RNA metabolism, oxidative stress responses, inflammatory responses, and immune system process (Figure 4). Thus, we selected top 53 upregulated DEGs with m^5^C gain that are highly associated with these processes to explore the potential influence of m^5^C in the lungs of mice with pulmonary fibrosis (Figure 5A). The IGV tracks of *Adipoq*, *Chi3l1*, *Mmp9*, and *Lcn2* are shown in Figure 5B as examples of alterations in m^5^C levels and gene expression. The tracks clearly exhibit m^5^C gain sites and the higher expression levels of these genes in PM_2.5_-exposed mice than in FA-exposed mice. Moreover, large changes in RNA abundance mediated by acquisition of m^5^C modifications were associated with pulmonary fibrosis induction (Figure 5C). Among the important genes selected (Figure 5A), *Lcn2* and *Mmp9* have been reported to participate in pulmonary fibrosis development [46], [47]. *Chi3l1* plays a major role in inflammation and is a potential target for prevention and treatment [48], whereas *Adipoq* is highly associated with the incidence of COPD [49]. The expression of these four key genes was also validated by RT-qPCR (Figure 5D).

These observations are consistent with a previous report showing the important role of *Lcn2* and *Mmp9* in inflammation and fibrosis through the IL-17 signaling pathway (Figure S4) [48], [49]. IL-17 induces inflammation by activating fibroblasts, endothelial cells, and epithelial cells [50]; it also enhances neutrophil recruitment to sites of inflammation [51]. In the present study, upregulation of *Mmp9* and *Lcn2* expression through m^5^C gain upon PM_2.5_ exposure may be critical in m^5^C-mediated fibrosis. Furthermore, m^5^C modification is potentially implicated in the pathogenesis of PM_2.5_-induced pulmonary fibrosis.

## Conclusion

In this study, we report the first epitranscriptomic RNA m^5^C profile in PM_2.5_-induced pulmonary fibrosis. Our study offers insight into the relationship between PM_2.5_-induced pulmonary fibrosis and an altered mRNA m^5^C methylome. Moreover, mRNA m^5^C gain probably negatively affects normal metabolic activity, including RNA metabolism, oxidative stress responses, inflammatory responses, and immune system processes by upregulating gene expression levels in lungs of PM_2.5_-exposed mice. The results suggest that mRNA m^5^C modification may play a critical role in the pathogenesis of PM_2.5_-induced pulmonary fibrosis. Altogether, the results provides valuable evidence for elucidating the mechanism of PM_2.5_ exposure-related lung pathogenesis and identifying novel potential biomarkers.

## Materials and methods

### Experimental animals

Four-week-old male C57BL/6 mice were purchased from Beijing Vital River Laboratory Animal Technology (Beijing, China). The mice were randomly assigned into the FA control group (*n* = 5) or PM_2.5_-exposed group (*n* = 5). Animals were either exposed to ambient PM_2.5_-polluted air or FA for 12 h/day, 7 days/week for 12 weeks (October 2017–January 2018) in a whole-body PM_2.5_ exposure system at the Zhongguancun Campus of the University of Chinese Academy of Sciences (UCAS), Beijing, China (N39°57′39.83″E116°20′10.97″). The exposure system included two separate chambers. In the FA chamber, ambient microparticles were removed by high-efficiency particulate air filter (Shanghai Liancheng Purification Equipment, Shanghai, China). In the PM_2.5_ chamber, ambient PM_2.5_ were collected with a swirler as previously reported [52]. The PM_2.5_ levels in the chamber were continually monitored and sampled daily. During the exposure period, the animals were maintained in exposure chamber with 12 h light/dark cycle (temperature: 21 °C–22 °C; humidity: 40%–60%) and given standard laboratory chow and sterile deionized water *ad libitum*.

### PM_2.5_ sampling and preparation

To evaluate the PM_2.5_ levels in the PM_2.5_ chamber, ambient PM_2.5_ was sampled during the exposure period every day. The distance between the sampling inlets and the PM_2.5_ chamber was 30 m. The PM_2.5_ particles were collected by Teflon coated filters (diameter = 90 mm, Whatman, St. Louis, MO) with medium-volume samplers (TH-150D II, Tianhong, Wuhan, China) at 100 l/min flow rate for 12 h (08:00–20:00) every day. Before and after sampling, the filters were equilibrated for 48 h at 30% relative humidity and room temperature (25 °C). Then the filters were weighed for calculation of ambient PM_2.5_ levels. The sampled PM_2.5_ was extracted by sonication into deionized water (18 MΩ·cm) according to the method we reported previously [53]. In brief, PM_2.5_ samples were sonicated for 0.5 h with the sonicator (Catalog No. KQ-700 V, Shumei, Kunshan, China). The PM_2.5_ suspension was stored at −80 °C prior to analysis.

The chemical properties of the PM_2.5_ were analyzed as described previously [53]. Briefly, the metal elements were extracted by acid digestion (HNO_3_: HF = 7:3). Then the solution measured by inductively coupled plasma mass spectrometry (ICP-MS, Elemental X7, Thermo Fisher Scientific, Waltham, MA). The water-soluble inorganic components were measured with ion chromatography (Dionex-600, Thermo Fisher Scientific). Organic carbon (OC) and elemental carbon (EC) were determined by thermal-optical analyzer (Sunset Laboratories, Tigard, OR). The concentrations of metal elements, inorganic components, and carbon of the PM_2.5_ samples are listed in Table S1.

### Tissue processing

After exposing to PM_2.5_, mice were anesthetized and sacrificed. The lung tissues were fixed using 4% paraformaldehyde solution, then embedded in paraffin. Lung sections (6 μm) were stained by H&E (Catalog No. G1005, Wuhan Servicebio Technology, Wuhan, China) or with a Masson trichrome stain kit (Catalog No. DC0033, Beijing Leagene, Beijing, China).

### Construction of the RNA-Seq and RNA-BisSeq libraries

Total RNA was extracted from mouse lungs with an RNAsimple Total RNA Kit (Catalog No. DP419, Tiangen, Beijing, China), and mRNA was isolated using a Dynabeads® mRNA Purification Kit (Catalog No.61006, Ambion, Waltham, MA). TURBO™ DNase (Catalog No. 61006, Ambion) treatment was used to eliminate DNA contamination at 37 °C for 30 min. Then, the mRNA was purified by ethanol precipitation. RNA extracted from the group of FA-exposed mice and that of PM_2.5_-exposed mice was pooled separately and used for construction of the RNA-Seq and RNA-BisSeq libraries.

The RNA-Seq libraries were constructed with a KAPA Stranded mRNA-Seq Kit (Catalog No. 07962207001, KAPA, Wilmington, MA). The RNA-BisSeq libraries were constructed based on the methods in a previous study with minor optimization [20]. Briefly, *in vitro-* transcribed mouse *Dhfr* mRNA, a methylation conversion control, was mixed with approximately 200 ng of purified mRNA at a ratio of 1:300. Then, the RNA was fragmented into ∼100-nt fragments in 10× RNA Fragmentation Reagent (Catalog No. AM8740, Ambion) at 90 °C (1 min), which was terminated by 10× RNA stop solution (Catalog No. AM8740, Ambion). After precipitation with 100% ethanol, the RNA pellet was resuspended in 100 µl of a bisulfite solution containing a 100:1 mixture of 40% sodium bisulfite (Catalog No. 13438, Sigma, St. Louis, MO) and 600 µM hydroquinone (pH 5.1; catalog No. H9003, Sigma), and heat-incubated at 75 °C for 4.5 h. Nanosep columns with 3 K Omega membranes (Catalog No. OD003C35, PALL Corporation, Port Washington, NY) were used to desalt the reaction mixture with centrifugation. The RNA pellet was washed with nuclease-free water followed by centrifugation for five times. Finally, the RNA was re-suspended in 75 μl of nuclease-free water and then incubated at 75 °C for 1 h with equal volume of 1 M Tris-HCl (pH 9.0) for desulfonation. The RNA was dissolved in 11 µl of RNase-free water after ethanol precipitation. After reverse transcription with SuperScript II Reverse Transcriptase (Catalog No. 18064014, Invitrogen, Waltham, MA) and ACT random hexamers, a KAPA Stranded mRNA-Seq Kit (Catalog No. 07962207001, KAPA) was used to perform the subsequent procedures according to the manufacturer’s instructions.

### Quantitative real-time PCR

Total RNA was isolated with an RNAsimple Total RNA Kit (Catalog No. DP419, Tiangen). cDNA was synthesized using a GoScript Reverse Transcription System (Catalog No. A5001, Promega, Madison, WI) according to the manufacturer’s instructions. The primer pairs used in this study are listed in Table S5. The gene expression levels were normalized to that of *ACTB*. RT-qPCR was performed as previously described [54]. *P* values were determined using two-tailed Student’s *t*-test. A difference with *P* < 0.05 was defined as significant.

### Western blotting analysis

Protein was extracted from the mouse lung tissues using RIPA lysis buffer containing PMSF (Catalog No. 97064-672, Amresco, Radnor, PA) and protease and phosphatase inhibitor cocktails (Catalog Nos. B14001 and B15001, Bimake, Houston, TX). The solution was centrifuged at 12,000*g* and 4 °C for 10 min, the supernatant was collected for further analysis. The expression of NSUN2 and β-actin in whole-cell lysates was analyzed by sodium dodecyl sulfate polyacrylamide gel electrophoresis (SDS-PAGE). Immunoreactive bands were detected with ECL reagents (Catalog No. 1705061, Bio-Rad, Berkeley, CA) according to the manufacturer’s instructions. The following antibodies were used: rabbit anti-NSUN2 (Catalog No. 20854-1-AP, Proteintech, Wuhan, China) and mouse β-actin (Catalog No. AF0003, Beyotime, Shanghai, China).

### Data analysis for high-throughput sequencing

RNA-Seq and RNA-BisSeq were performed using an Illumina HiSeq 2500 platform with a paired-end read length of 150 bp. The adaptor sequences were trimmed off and low-quality bases were removed using Trimmomatic (version 0.33) [55]. For RNA-Seq, the remaining reads with lengths greater than 35 nt were used for the alignment with the mouse reference genome (version mm10) with TopHat (version 2.1.1, default parameters) [56]. Uniquely mapped reads (*q* ≥ 20) were used for the downstream analysis (Table S2). DEGs were detected with the DEGseq package in the R language [57]. Genes with FC > 1.2 and FDR < 0.05 were defined as significantly up- or downregulated genes. For RNA-BisSeq, reads that contained more than three sequential Cs were removed to reduce the number of false positives [58], [59], and the resulting reads were then aligned to reference genomes with meRanT align (meRanTK version 1.2.0) [60] with parameters: -fmo -mmr 0.01. The m^5^C sites were called by meRanCall (meRanTK, version 1.2.0) [60] with parameters: -mBQ 20 -mr 0 and only sites with coverage depth ≥ 10, methylation level ≥ 0.1, methylated cytosine depth ≥ 2 were considered credible. BEDTools’ intersectBed (version 2.26.0) [61] was used to annotate the m^5^C sites. mRNAs with m^5^C gain or loss were defined as mRNAs that were specifically modified in the PM_2.5_ exposure or control samples. IGV (IGVTools, version 2.3.8) [62] was used for visualization.

To explore the essential role of m^5^C modification in PM_2.5_-induced pulmonary fibrosis in mice, DEGs were separated into four groups based on m^5^C gain or loss and upregulation or downregulation of gene expression (FC > 1.2, FDR < 0.05). Only genes with reads per kilobase per million reads (RPKM) >1 in at least one sample and mRNA with FC > 1.5 were subjected to downstream analysis. The signaling network in lungs of mice exposed to PM_2.5_ was assessed with KEGG Mapper (https://www.kegg.jp/kegg/mapper.html). DAVID (version 6.8, http://david.abcc.ncifcrf.gov/) and Metascape (http://metascape.org) were used to perform GO analysis. GO terms with *P* < 0.05 (hypergeometric test) were considered statistically significant (Table S3). DEGs from the four groups mentioned above were used to perform PPI network analysis. PPI network was generated using the Search Tool for the Retrieval of Interacting Genes (STRING) database [63] (Table S4) and visualized with Cytoscape (version 3.6.0) [64].

Promoter is defined as the region ±2 kb around the transcription start site [45]. Prediction of TFs that potentially bind to the promoter sequence of *Nsun2*, was performed on https://biogrid-lasagna.engr.uconn.edu/lasagna_search/.

## Ethical statements

The animal studies were performed under the guidance of laboratory animal care (NIH publication no. 85-23, revised 1985) and approved by the University of Chinese Academy of Sciences Animal Care and Use Committee.

## Data availability

The RNA-Seq and RNA-BisSeq data have been uploaded to the Gene Expression Omnibus database (GEO: GSE122493), and to the Genome Sequence Archive [65] at the National Genomics Data Center, Beijing Institute of Genomics (BIG), Chinese Academy of Sciences/China National Center for Bioinformation (GSA: CRA001230 with BioProject ID: PRJCA001116), and are publicly accessible at https://bigd.big.ac.cn/gsa/.

## Authors’ contributions

WD and YGY conceived this project. HL, XH, ZZ, and WY performed the experiments and analyzed the data. CW and FZ contributed to animal care and technical supports. XL performed the physiochemical characterization of PM_2.5_ analysis. XH and BS performed bioinformatics analysis. XH, HL, ZZ, YZ, and GJ wrote the manuscript with the help of all authors. All authors read and approved the final manuscript.

## Competing interests

The authors have declared no competing interests.

## Supplementary material

Supplementary Figure S1 **Distribution features of mRNA m^5^C in the lungs of FA and PM_2.5_-exposed mice A.** Heatmap illustrating the mRNA expression levels and m^5^C modification of several pulmonary fibrosis-related genes in the lung. The color bar represents the normalized RPKM of the genes. The blue and white boxes represent m^5^C-modified and unmodified genes, respectively. **B.** Pie chart showing the transcriptome-wide distribution of mRNA m^5^C sites. **C.** Sequence frequency logo for the sequences proximal to mRNA m^5^C sites (generated using WebLogo). **D.** RNA-BisSeq results revealing the numbers of m^5^C-modified mRNAs among different chromosomes. MT, mitochondria.

Supplementary Figure S2 **PM_2.5_ exposure leads to altered gene expression in mouse lungs A.** Scatter plots showing the coverage of genes with m^5^C gain and loss in lung samples from FA-exposed and PM_2.5_-exposed mice. The Pearson correlation coefficient (R) is shown in the top left corner. **B.** Volcano plots displaying enrichment of DEGs in lung samples from PM_2.5_-exposed mice compared to FA-exposed mice. The numbers of upregulated (blue) or downregulated (purple) DEGs (FC > 1.2, FDR < 0.05) are shown. The vertical dashed lines indicate the FC cut-off (1.2), whereas the horizontal dashed lines indicate the FDR cut-off (0.05). **C.** Representative GO biological process categories enriched for the upregulated genes. **D.** Representative GO biological process categories enriched for the downregulated genes. The color bar represents the −log_10_*P* value.

Supplementary Figure S3 **Bubble diagram of GO biological process categories enriched for DEGs with m^5^C gain or loss** The pink circle represents the −log_10_*P* value.

Supplementary Figure S4 **Signaling network of m^5^C-modified Mmp9 and Lcn2 in pulmonary fibrosis mice** The network was produced using KEGG Mapper (). *Mmp9* and *Lcn2* were marked in red and yellow, respectively.
