# Supplementary figures and images for: Epitranscriptomic 5-Methylcytosine Profile in PM2.5-induced Mouse Pulmonary Fibrosis
Source: Genomics Proteomics Bioinformatics. 2020 Mar 3;18(1):41–51. doi: 10.1016/j.gpb.2019.11.005 (PMC7393542; doi:10.1016/j.gpb.2019.11.005)

**A**

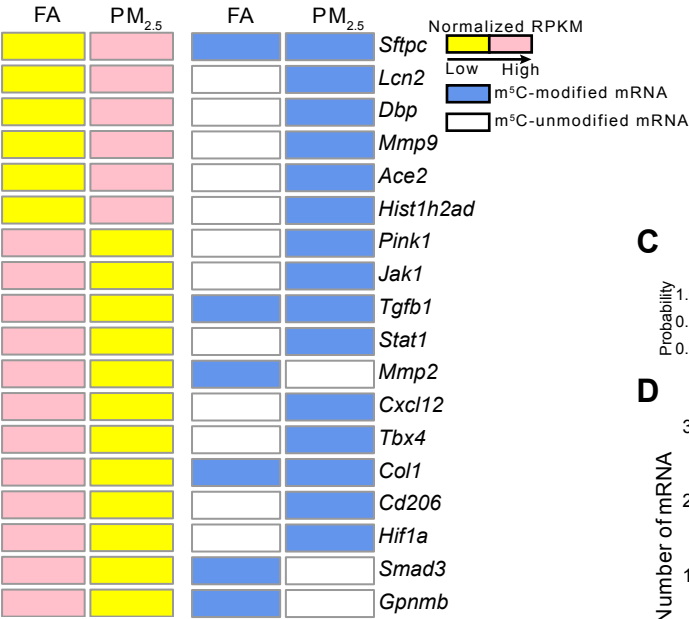

**B**

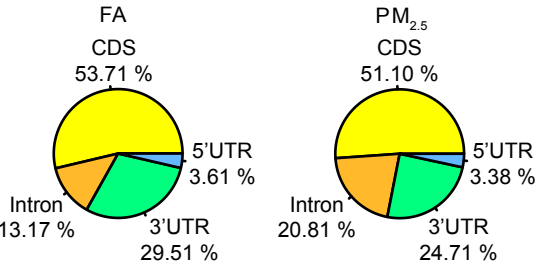

**C**

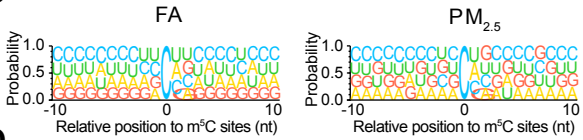

**D**

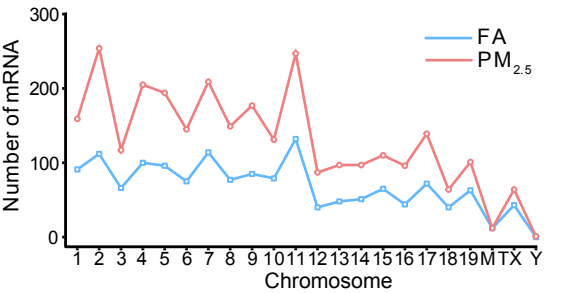

Supplement: Supplementary Figure S1 — Distribution features of mRNA m5C in the lungs of FA and PM2.5-exposed mice A. Heatmap illustrating the mRNA expression levels and m5C modification of several pulmonary fibrosis-related genes in the lung. The color bar represents the normalized RPKM of the genes. The blue and white boxes represent m5C-modified and unmodified genes, respectively. B. Pie chart showing the transcriptome-wide distribution of mRNA m5C sites. C. Sequence frequency logo for the sequences proximal to mRNA m5C sites (generated using WebLogo). D. RNA-BisSeq results revealing the numbers of m5C-modified mRNAs among different chromosomes. MT, mitochondria. [file mmc1.pdf]

**A**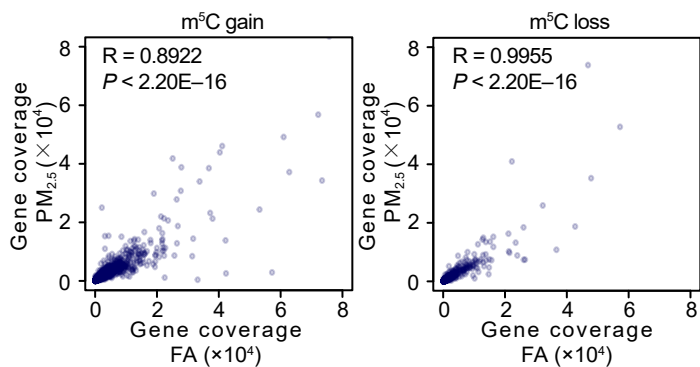**B**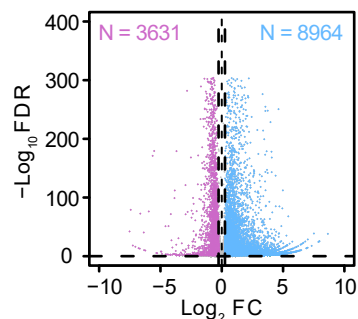**C**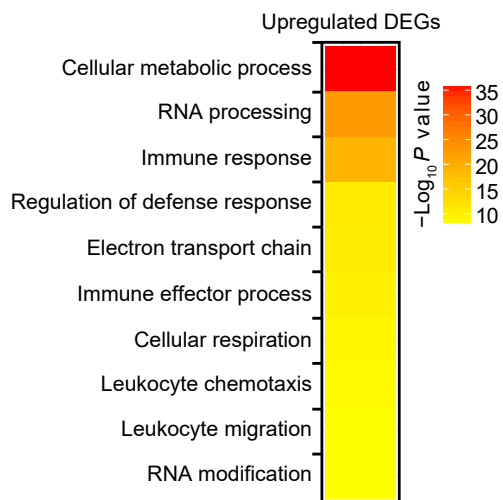**D**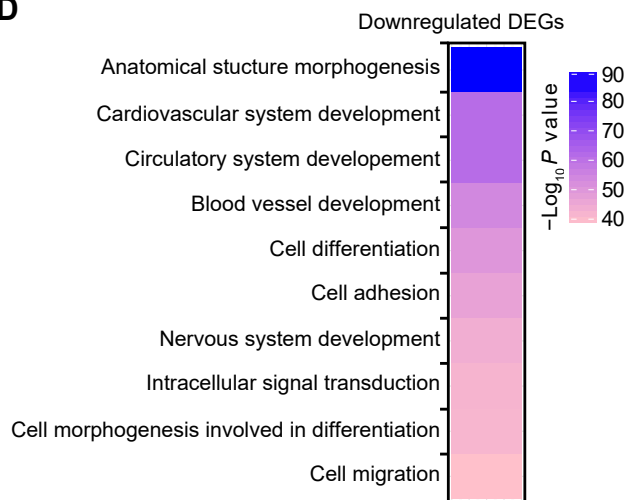

Supplement: Supplementary Figure S2 — PM2.5 exposure leads to altered gene expression in mouse lungs A. Scatter plots showing the coverage of genes with m5C gain and loss in lung samples from FA-exposed and PM2.5-exposed mice. The Pearson correlation coefficient (R) is shown in the top left corner. B. Volcano plots displaying enrichment of DEGs in lung samples from PM2.5-exposed mice compared to FA-exposed mice. The numbers of upregulated (blue) or downregulated (purple) DEGs (FC > 1.2, FDR < 0.05) are shown. The vertical dashed lines indicate the FC cut-off (1.2), whereas the horizontal dashed lines indicate the FDR cut-off (0.05). C. Representative GO biological process categories enriched for the upregulated genes. D. Representative GO biological process categories enriched for the downregulated genes. The color bar represents the −log10P value. [file mmc2.pdf]

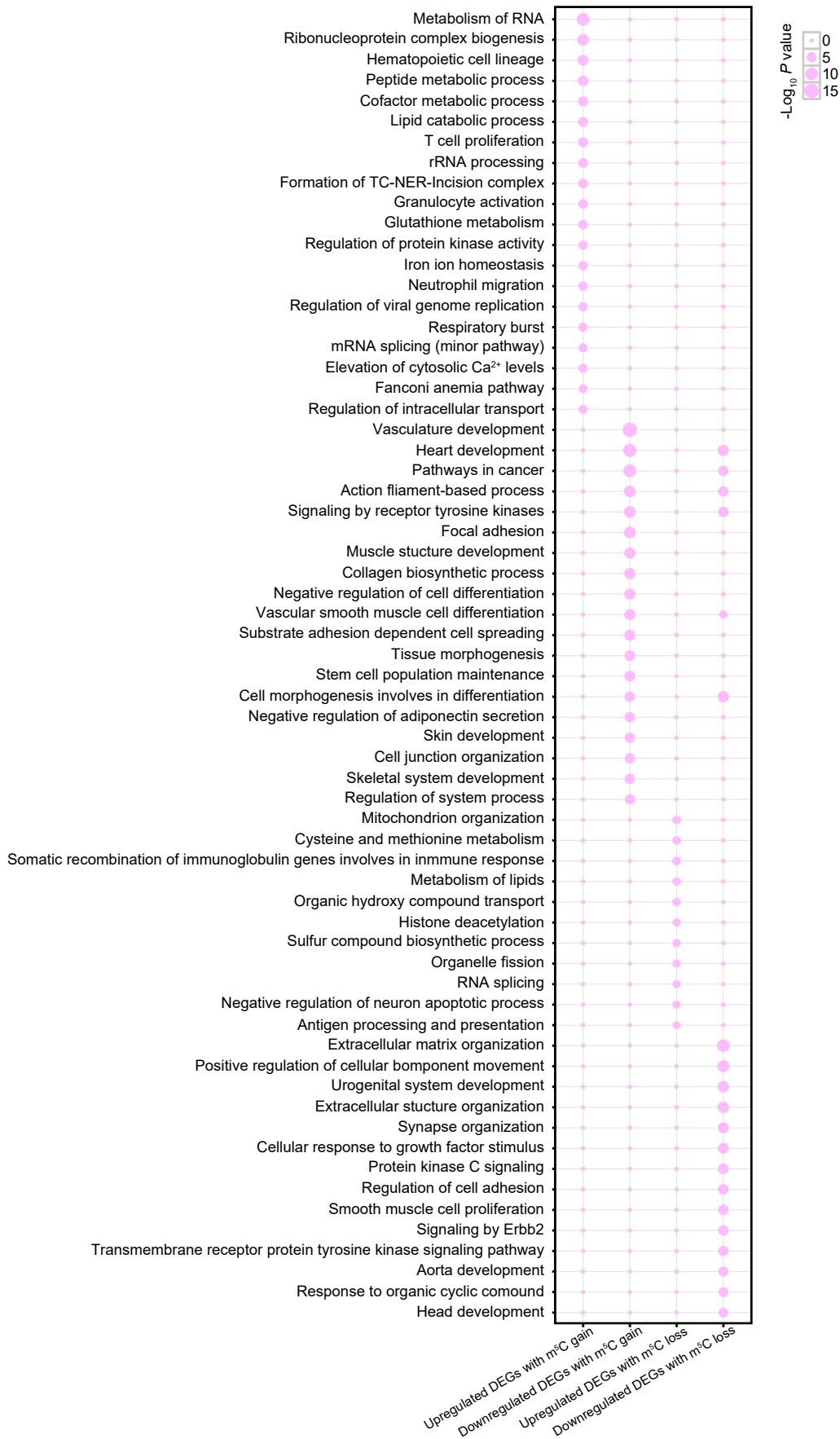

Supplement: Supplementary Figure S3 — Bubble diagram of GO biological process categories enriched for DEGs with m5C gain or loss The pink circle represents the −log10P value. [file mmc3.pdf]

# IL-17 SIGNALING PATHWAY

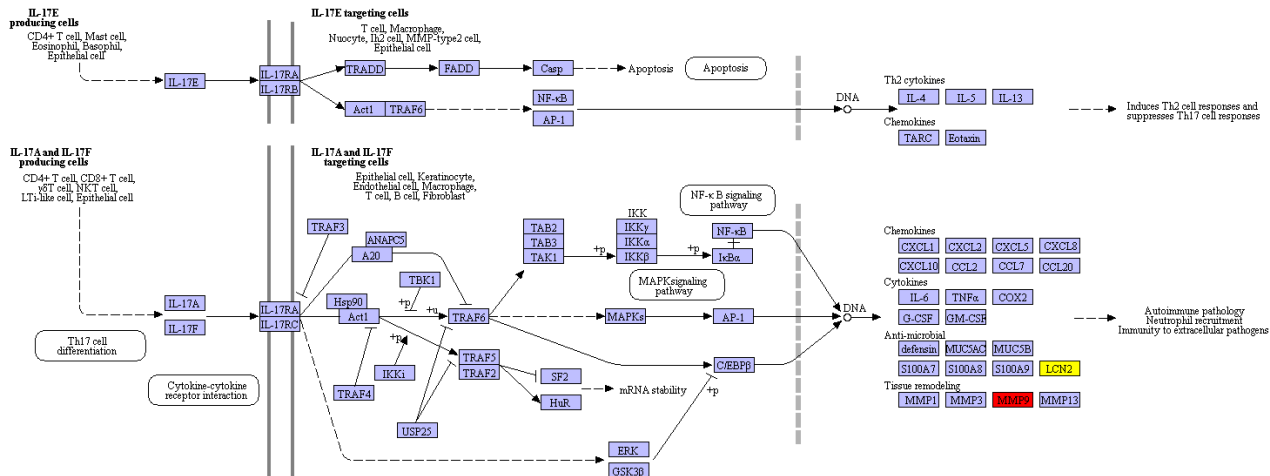

Supplement: Supplementary Figure S4 — Signaling network of m5C-modified Mmp9 and Lcn2 in pulmonary fibrosis mice The network was produced using KEGG Mapper (). Mmp9 and Lcn2 were marked in red and yellow, respectively. [file mmc4.pdf]
