# Supplementary material for: Epitranscriptomic 5-Methylcytosine Profile in PM2.5-induced Mouse Pulmonary Fibrosis
Source: Genomics Proteomics Bioinformatics. 2020 Mar 3;18(1):41–51. doi: 10.1016/j.gpb.2019.11.005 (PMC7393542; doi:10.1016/j.gpb.2019.11.005)
Supplement: Supplementary Table S1 [file mmc5.docx]

**Table S1 Chemical composition analysis of the PM_2.5_ samples**

|  | **Component** | **Concentration (μg/mg)** |
| --- | --- | --- |
| Metal elements | Ca | 0.38 ± 0.0006 |
|  | K | 0.15 ± 0.0001 |
|  | Na | 0.56 ± 0.0001 |
|  | Mg | 0.65 ± 0.0001 |
|  | Ti | 1.27 ± 0.01 |
|  | Zn | 1.55 ± 0.046 |
|  | Pb | 0.23 ± 0.007 |
|  | Mn | 0.52 ± 0.016 |
|  | Cu | 0.20 ± 0.022 |
|  | V | 0.044 ± 0.002 |
|  | Ba | 0.51 ± 0.018 |
|  | Cr | 0.060 ± 0.005 |
|  | As | 0.063 ± 0.002 |
|  | Ni | 0.026 ± 0.002 |
|  | Sr | 0.22 ± 0.006 |
|  | Cd | 0.011 ± 0.004 |
|  | Mo | 0.010 ± 0.002 |
|  | Cs | 0.004 ± 0.0002 |
|  | Co | 0.007 ± 0.0003 |
| Inorganic ions | SO_4_^2−^ | 508.17 ± 0.03 |
|  | NO_3_^−^ | 1315.83 ± 0.08 |
|  | NH_4_^+^ | 665.37 ± 0.03 |
|  | Cl^−^ | 298.10 ± 0.01 |
| Carbon | OC | 100.12 ± 1.66 |
|  | EC | 22.38 ± 0.46 |
|  | OC/EC | 4.47 ± 0.17 |

*Note*: OC, organic carbon; EC, elemental carbon.
