# Supplementary material for: Epitranscriptomic 5-Methylcytosine Profile in PM2.5-induced Mouse Pulmonary Fibrosis
Source: Genomics Proteomics Bioinformatics. 2020 Mar 3;18(1):41–51. doi: 10.1016/j.gpb.2019.11.005 (PMC7393542; doi:10.1016/j.gpb.2019.11.005)
Supplement: Supplementary Table S2 [file mmc6.docx]

**Table S2 Summary of the sequencing sample information**

| **Sequencing type** | **Sample** | **No. of total reads** | **No. of clean reads** | **No. of uniquely-mapped reads** | **Conversion rate** |
| --- | --- | --- | --- | --- | --- |
| RNA-BisSeq | C-12W-BS | 225,959,344 | 177,973,176 | 80,088,482 | 99.37% |
|  | PM-12W-BS | 176,070,088 | 137,339,808 | 60,189,948 | 99.25% |
| RNA-Seq | C-12W | 53,812,509 | 44,379,648 | 30,466,628 | - |
|  | PM-12W | 53,168,021 | 46,268,952 | 30,000,788 | - |

*Note*: C, FA-exposed control; PM, PM_2.5_; W, weeks.
