# Supplementary material for: Epitranscriptomic 5-Methylcytosine Profile in PM2.5-induced Mouse Pulmonary Fibrosis
Source: Genomics Proteomics Bioinformatics. 2020 Mar 3;18(1):41–51. doi: 10.1016/j.gpb.2019.11.005 (PMC7393542; doi:10.1016/j.gpb.2019.11.005)
Supplement: Supplementary Table S5 [file mmc9.docx]

**Table S5 Primers used for RT-qPCR**

| **GenBank accession No.** | **Primer name** | **Direction** | **Primer sequence (5'-3')** |
| --- | --- | --- | --- |
| NM_009605.5 | *Adipoq*_FP | Forward | GAAGCCGCTTATGTGTATCGC |
|  | *Adipoq*_RP | Reverse | GAATGGGTACATTGGGAACAGT |
| NM_020582.2 | *Atp5j2*_FP | Forward | TGCCGAGCTGGATAATGATGC |
|  | *Atp5j2*_RP | Reverse | ACCATGCTAATCCCCGAGATG |
| NM_013795.5 | *Atp5l*_FP | Forward | GAGAAGGCACCGTCGATGG |
|  | *Atp5l*_RP | Reverse | ACACTCTGAATAGCTGTAGGGAT |
| NM_007512.4 | *Atpif1*_FP | Forward | GGTGTCTGGGGTATGAAGGTC |
|  | *Atpif1*_RP | Reverse | CCTTTTCTCGTTTTCCGAAGGC |
| NM_007695.3 | *Chi3l1*_FP | Forward | GTACAAGCTGGTCTGCTACTTC |
|  | *Chi3l1*_RP | Reverse | ATGTGCTAAGCATGTTGTCGC |
| NM_010208.4 | *Fgr*_FP | Forward | CAGAGGCTATGTTCCCAGCAA |
|  | *Fgr*_RP | Reverse | CTGATCTTTCCGAAGTACCACTC |
| NM_010548.2 | *Il10*_FP | Forward | GCTCTTACTGACTGGCATGAG |
|  | *Il10*_RP | Reverse | CGCAGCTCTAGGAGCATGTG |
| NM_010552.3 | *Il17a*_FP | Forward | TTTAACTCCCTTGGCGCAAAA |
|  | *Il17a*_RP | Reverse | CTTTCCCTCCGCATTGACAC |
| NM_031168.2 | *Il6*_FP | Forward | TAGTCCTTCCTACCCCAATTTCC |
|  | *Il6*_RP | Reverse | TTGGTCCTTAGCCACTCCTTC |
| NM_001048177.3 | *Jak2*_FP | Forward | GGAATGGCCTGCCTTACAATG |
|  | *Jak2*_RP | Reverse | TGGCTCTATCTGCTTCACAGAAT |
| NM_008491.1 | *Lcn2*_FP | Forward | GGGAAATATGCACAGGTATCCTC |
|  | *Lcn2*_RP | Reverse | CATGGCGAACTGGTTGTAGTC |
| NM_013599.4 | *Mmp9*_FP | Forward | CTGGACAGCCAGACACTAAAG |
|  | *Mmp9*_RP | Reverse | CTCGCGGCAAGTCTTCAGAG |
| NM_145354.5 | *Nsun2*_FP | Forward | ACACTGAGAATCACTGGGTACA |
|  | *Nsun2*_RP | Reverse | CCAGCTTAGTGGTTGTGGAACT |
| NM_013612.2 | *Slc11a1*_FP | Forward | GTGGGCTCAGATATGCAGGAA |
|  | *Slc11a1*_RP | Reverse | GCGCAAACCATAGTTATCCAAGA |
| NM_011577.2 | *Tgfb1*_FP | Forward | CTCCCGTGGCTTCTAGTGC |
|  | *Tgfb1*_RP | Reverse | GCCTTAGTTTGGACAGGATCTG |
| NM_011593.2 | *Timp1*_FP | Forward | GCAACTCGGACCTGGTCATAA |
|  | *Timp1*_RP | Reverse | CGGCCCGTGATGAGAAACT |
| NM_011662.3 | *Tyrobp*_FP | Forward | CCCAAGATGCGACTGTTCTTC |
|  | *Tyrobp*_RP | Reverse | GTCCCTTGACCTCGGGAGA |

*Note*: Adipoq, adiponectin, C1Q and collagen domain-containing; Atp5j2, ATP synthase, H^+^-transporting mitochondrial F0 complex subunit F2; Atp5l, ATP synthase, H^+^-transporting mitochondrial F0 complex subunit G; Atpif1, ATPase inhibitory factor 1; Chi3l1, chitinase-like 1; Fgr, FGR proto-oncogene, Src family tyrosine kinase; Il10, interleukin 10; Il17a, interleukin 17A; Il6, interleukin 6; Jak2, janus kinase 2; Lcn2, lipocalin 2; Mmp9, matrix metallopeptidase 9; Nsun2, NOL1/NOP2/Sun domain family, member 2; Slc11a1, solute carrier family 11; Tgfb1, transforming growth factor, beta 1; Timp1, tissue inhibitor of metalloproteinase 1; Tyrobp, TYRO protein tyrosine kinase binding protein.
